# Methylcellulose Based Thermally Reversible Hydrogel System for Tissue Engineering Applications

**DOI:** 10.3390/cells2030460

**Published:** 2013-06-25

**Authors:** Sreedhar Thirumala, Jeffrey M. Gimble, Ram V. Devireddy

**Affiliations:** 1 BioTechnology LLC, 1102 Indiana Avenue Indianapolis, IN 46202, USA; 2 Stem Cell Biology Laboratory, Pennington Biomedical Research Center, Louisiana State University System, Baton Rouge, LA 70808, USA; 3 Laboratory, Department of Mechanical Engineering, Louisiana State University, Baton Rouge, LA 70803, USA

**Keywords:** cell sheet engineering, temperature-responsive polymers, adult stem cells, scaffolds

## Abstract

The thermoresponsive behavior of a Methylcellulose (MC) polymer was systematically investigated to determine its usability in constructing MC based hydrogel systems in cell sheet engineering applications. Solution-gel analyses were made to study the effects of polymer concentration, molecular weight and dissolved salts on the gelation of three commercially available MCs using differential scanning calorimeter and rheology. For investigation of the hydrogel stability and fluid uptake capacity, swelling and degradation experiments were performed with the hydrogel system exposed to cell culture solutions at incubation temperature for several days. From these experiments, the optimal composition of MC-water-salt that was able to produce stable hydrogels at or above 32 °C, was found to be 12% to 16% of MC (Mol. wt. of 15,000) in water with 0.5× PBS (~150mOsm). This stable hydrogel system was then evaluated for a week for its efficacy to support the adhesion and growth of specific cells in culture; in our case the stromal/stem cells derived from human adipose tissue derived stem cells (ASCs). The results indicated that the addition (evenly spread) of ~200 µL of 2 mg/mL bovine collagen type -I (pH adjusted to 7.5) over the MC hydrogel surface at 37 °C is required to improve the ASC adhesion and proliferation. Upon conﬂuence, a continuous monolayer ASC sheet was formed on the surface of the hydrogel system and an intact cell sheet with preserved cell–cell and cell–extracellular matrix was spontaneously and gradually detached when the grown cell sheet was removed from the incubator and exposed to room temperature (~30 °C) within minutes.

## 1. Introduction

Long-established tissue engineering methods routinely used either the injection of isolated single cell suspensions or biodegradable scaffolds to support the growth and development of cells into tissues [1]. During the past decade, significant progress has been made in using cellularized extracellular scaffolds to regenerate various tissues including bone, cartilage, heart, blood vessel, nerve, liver, and many other tissues [2,3,4,5,6,7,8,9]. However, most of the conventional scaffolds are complicated in construction and do not allow sufficient cell migration to establish adequate cell–cell adhesion, cell–cell communication and cell–extracellular matrix (ECM) which are all critically important tissue level functions [10]. Therefore, high density cell seeding is commonly utilized in static and dynamic cultures inside bioreactors to establish adequate cell–extracellular matrix (ECM) and cell–cell interactions [11,12,13,14,15,16,17]. The dissemination of seeded cells within the scaffold plays a crucial role in determining the progression of tissue formation to establish 3D cell culture [9,10]. Furthermore, due to the lack of vascularization, the cells located in the interior of the scaffold rely on diffusion for solute transport, and thus, the current methods of scaffold based cell seeding methods are compromised by the depletion of nutrients by cells located near the outer surface [9,10]. 

To overcome some of the drawbacks associated with traditional scaffold based tissue engineering, cell sheet engineering has been developed as an alternative approach for tissue engineering [18,19,20,21,22,23]. Cell sheet engineering has the advantage of eliminating the use of biodegradable scaffolds. Using temperature-responsive poly(N-isopropylacrylamide) (PIPAAm) coated Tissue Culture Poly-Styrone (TCPS) dishes, cultured cells were successfully harvested as intact sheets by simple temperature changes, thereby avoiding the use of proteolytic enzymes [1]. In a recent study, a novel method, using a thermoresponsive hydrogel coated on TCPS dishes, was developed for harvesting living cell sheets [24]. The hydrogel was prepared by pouring aqueous methylcellulose blended with distinct salts on TCPS dishes at room temperature and subsequently gelled at 37 °C for cell culture. Methylcellulose (MC) with a viscosity of 4000cP at 2% solution was used to form gels at physiologically relevant temperatures [24]. However, the procedure described was complex and do not consistently produce stable hydrogels as the MC formulation used was too viscous to be manipulated easily [24]. Besides, due to the high viscosity of the MC formulation, it was difficult to spread MC hydrogel evenly onto tissue culture dish and therefore resulted in non-uniform coating and a subsequent unstable hydrogel system. However, for cell sheet engineering applications, MC hydrogel systems generated have to be consistently stable as the time of culture is increased. To address the shortcomings associated with the earlier MC based cell sheet engineering technology [24] we have developed a modified MC-collagen hydrogel method to create cell sheets, specifically tailored to work with adipose tissue derived stromal/stem cells (ASCs) and for the creation of multi-dimensional cell sheets. As described in the present study, by undertaking a systematic study of the thermal (gelation, degradation and swelling) properties of MC solutions of varying molecular weights, salt concentration, polymer concentration, we were able to discover the optimal composition of MC-aqueous solutions that will form stable gels at experimentally viable temperatures of ~32 °C. A brief description of our experiments in developing/characterizing/fabricating a hydrogel system that is suitable for cell culture and bench-top lab experiments, is provided.

## 2. Experimental Section

Methylcellulose (MC), with the trade names of M7140, M0262 and M0512 were obtained from Sigma Chemicals (Sigma Chemical Company, USA). According to the product specifications, the number-average molecular weights of M7140, M0262 and M0512 are 15,000, 41,000 and 88,000 respectively. Table 1 shows the formulations tested in this study when compared to Chen *et al.* [24]. Type I collagen solution (3 mg/mL Ultra Pure bovine) was purchased from Sigma-Aldrich (Sigma Chemical Company, USA). 

**Table 1:** Comparison of the formulations tested in this study with that of Chen *et al*. [24].

	Present Study			Chen *et al.* [24]
**MC**	M0512 (MW = 88,000)	M0262 (MW = 41,000)	M7140 (MW = 15,000)	Methocel 64630 (MW = 77000-94000)
**MC** **Concentration (%)**	1,2,3,4,5,6	2,4,6,8,10	2,4,6,8,10,12,14,16	1,2,3,4
**Salts**	D-PBS	D-PBS	D-PBS	NaCl, Na_2_SO_4_, Na_3_-PO_4_, D-PBS
**Salt Concentrations (Moles)**	0M,0.3M,0.6M,0.9M,1.2M,1.5M,1.8M	0M, 0.15M, 0.3M	0M, 0.15M, 0.3M	0M,0.1M,0.2M,0.4M,0.6M,0.8M,1.0M

### 2.1. Preparation of MC-Aqueous Solutions

MC solutions were formed by a dispersion technique, as described in the product manual. Initially, the solvent consisted of pure distilled water. Approximately half of the required volume of solvent was heated to ~90 °C before the MC powder was stirred in and thoroughly wetted. The remainder of the solvent volume at RT (~30 °C) was then added to the heated-MC mixture and gently stirred. As the temperature was lowered, the polymer became water-soluble, forming a clear solution. Once the entire polymer solution was brought to RT, the solution was lightly stirred/agitated for a further 30 min and the solutions were allowed to equilibrate overnight in a 4 °C refrigerator. Prior to use, the solutions were allowed to slowly equilibrate (~couple of hours) with the room temperature by placing them in a sterile environment.

Table 2 lists the composition of the various MC-water mixtures investigated in this study. Note that as the molecular weight of the MC increases, the % of MC that can be dissolved in water decreases to a maximum of 6% for MC M0512 (Mol. wt of 88,000) to a maximum of 10% for MC M0262 (Mol. wt of 41,000) and to a maximum of 16% for MC M7140 (Mol. wt of 15,000). Additional experiments were also conducted by adding salts (increasing the osmolality) to the MC-water system. Specifically, for all the MC-water the solutions the osmolality was adjusted to either 0.5× (0.15M) or 1× (0.3M) iso-osmolality. 

**Table 2:** Gelation Temperature of the Methylcellulose (MC)-water solutions as a function of molecular weight and concentrations.

MC Concentration (%)	Gel Point from Calorimetry Experiments (°C)		
	M0512 (mol. wt. = 88,000)	M0262 (mol. wt. = 41,000)	M7140 (mol. wt. = 15,000)
**2**	53.1 (±0.8)	52.5 (±0.3)	50.8 (±0.7)
**4**	52.2 (±1.3)	48.2 (±1.1)	46.4 (±1.9)
**6**	48.2 (±1.0)	45.8 (±1.4)	44.9 (±1.5)
**8**	—	43.2 (±1.4)	44.1 (±0.9)
**10**	—	41.7 (±2.0)	40.1 (±1.2)
**12**	—	—	37.6 (±0.8)
**14**	—	—	34.6 (±1.9)
**16**	—	—	31.1 (±1.5)

### 2.2. Calorimetric Characterization of Aqueous MC Solutions

The gelation temperature of the various MC-aqueous solutions was investigated using a differential scanning calorimeter (DSC, Pyris Diamond, Perkin-Elmer, Shelton, CT, USA), as described earlier [25,26]. DSC has been routinely used to detect endothermic heat of dehydration of polymeric materials. Briefly, 15 to 20 µL of the MC-water solution was placed in the sample pan and heated from 10 to 90 °C at a heating rate of 10 °C/min. The reference sample pan was filled with 15 µL of deionized water to increase the sensitivity of the measurements [25,26]. To further assure ourselves of the correlation between the endothermic peaks from the DSC thermogram and the gelation of methylcellulose, the gelation was also assessed and validated using the qualitative tube inversion method [24,27]. In this method it was assumed that a sample having a yield stress (gel) will not flow whereas a viscous but inelastic sample (sol) will show appreciable flow [24,27]. Gel formation temperature values for the MC concentration series obtained from both DSC and tube inversion measurements were found to be statistically equivalent indicating the DSC endothermic peak was the representation of gel formation during heating (data not shown).

### 2.3. Preparation of the MC Hydrogel Coated Tissue Culture Polystyrene (TCPS) Dish

**Figure 1:**
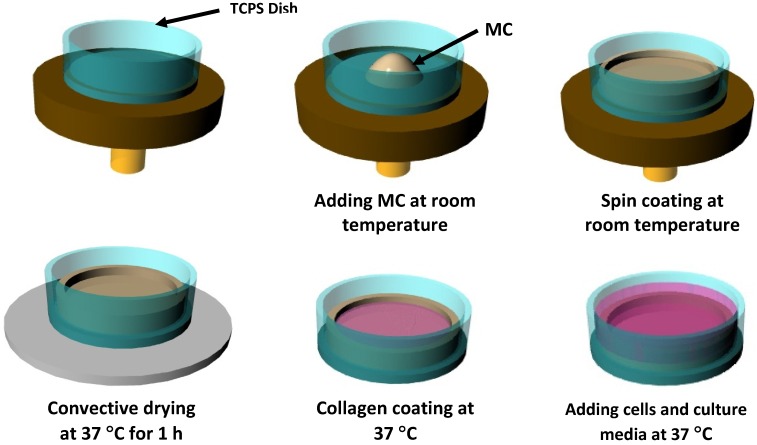
Schematic illustration of the preparation of methylcellulose (MC)-Collagen hydrogel system using spin coating procedure. Approximately 400 µL of the aqueous MC solution was poured into the center of each TCPS dish at room temperature. Spin coating was used as described in the Materials and Methods to evenly spread a thin transparent layer of MC on the TCPS dish. After a convective drying at 37 °C for one hour, a thin layer of collagen type 1 was evenly spread using a sterile cotton swab for improving cell adherence to MC hydrogel system.

The pre-selected MC-aqueous solution that had gelation temperatures below 37 °C were used to coat TCPS dishes (Falcon 3653, diameter 35 mm, Becton Dickinson Labware, Franklin Lakes, NJ, USA). An amount of approximately 400 µL of the chosen MC-aqueous solution was poured into the center of each TCPS dish at RT. Spin coating (SCS Special Coating Systems, Inc. MN; P6000 Spin coater; Model No. P0204-A) was employed to evenly spread a thin transparent layer of MC on the TCPS dish. All the MC coatings were prepared by spinning the sample at 850 rpm for 12 s (for 4% MC M0512 or 8% MC M0262 or 14%–16% MC M7140 in 0×, 0.5× and 1× D-PBS solutions). Subsequently, the MC coated dish was incubated at 37 °C for 1 h, at which time an opaque and gelled MC hydrogel layer was formed on the dish (See Figure 1). 

### 2.4. *In vitro* Degradation, Swelling and Osmotic Stability of MC Gels

The initial weight of the gelled MC hydrogel layer was measured and denoted as “MC weight”. To investigate the degradation and swelling behavior of the gelled MC hydrogel layer, approximately 2 mL of cell culture media, (DMEM-F12 and 10% fetal bovine serum) was added to the TCPS dishes. The weight of the gelled MC hydrogel layer immediately (~10 s) after the addition of cell incubation media was also measured and denoted as “Wet MC weight @ t = 0”. The TCPS dishes with the 2 mL of media were incubated at 37 °C for several days; subsequently, the cell culture media was removed from the TCPS dishes at various time (t) points (1, 4, 7 and 10 days) of incubation and the weight of the MC hydrogel was recorded and denoted as “Wet MC weight @ t”. The MC hydrogel layers were then dried overnight in a convective heater (VWR Scientific, Bridgeport, NJ, USA; 1602 HAFO series) at 70 °C, and the dried MC weight was recorded and denoted as “Dried MC weight @ t”. Swelling and degradation were quantified, as a function of time (t), using the following relationships:

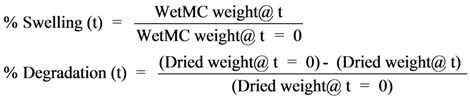



Further, to evaluate whether the salts blended in the MC hydrogel would leach out with time during *in vitro* culture, the osmolality of the loaded media was monitored with time using an osmometer (Model 3300, Advanced Instruments, Inc., Norwood, MA, USA). An uncoated TCPS dish loaded with the same media was used as control. 

### 2.5. Culture of ASCs on MC Coated TCPS Dishes

To improve the cell adhesion properties, the MC hydrogel surface was further coated by spreading with 200 µL of aqueous type I collagen (0.5 mg/mL, bovine dermis collagen, Sigma Chemical Co, USA). Since only alkaline collagen forms a gel network at physiological temperatures, the collagen solution was adjusted to a pH of 7.5 using NaOH and HCl and 10× PBS. For further dilutions 10× (3 M) phosphate buffered saline, PBS (Sigma Chemical Company, USA) was used. Adipose stem cells were isolated from human adipose tissue as described elsewhere [28,29,30,31,32,33] and obtained with appropriate institutional approval (PBRC #23040). Passage 0 ASCs were added to the MC coated dish along with the stromal media containing 90% DMEM/F-12, 10% fetal bovine serum and 1% penicillin-streptomycin. Care was taken to add media at 37 °C. Cell attachment and growth were observed daily using a light microscope (E600, Nikon, Tokyo, Japan) for 7 days. A collagen-coated TCPS dish was used as control. 

## 3. Results and Discussion

### 3.1. Effect of Molecular Weight

**Figure 2:**
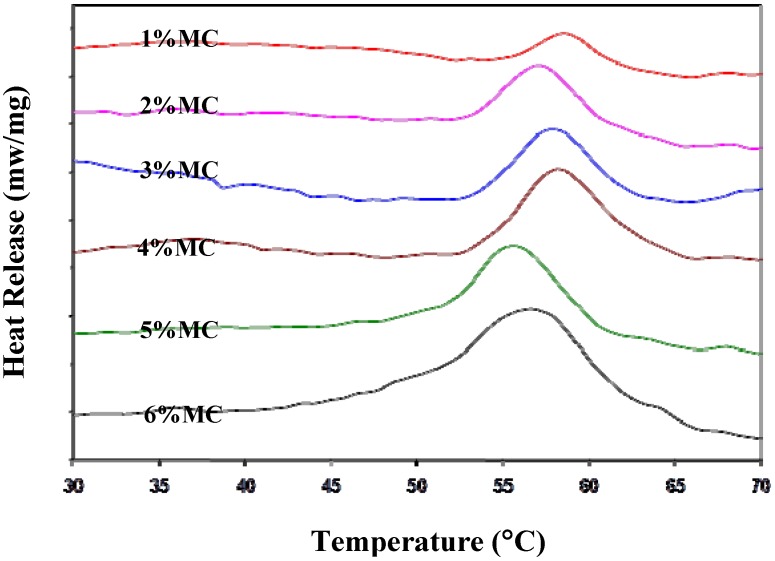
A series of differential scanning calorimeter (DSC) thermograms showing the incipient gelation temperature of MC M0512 (Mol. wt of 88,000) at various concentrations (1, 2, 3, 4, 5, and 6%) in 1× PBS solution. The thermograms from top to bottom represent increasing concentrations of MC in solution. For the ease of visualization, the thermograms for all the concentrations were displaced by a stepwise amount to prevent overlapping of the curves. As shown in Figure 2, increasing the amount of MC in solution not only increases the endothermic peak height but it also makes it broader. This suggests that the total energy absorbed during heating of the MC-solutions is dependent upon the concentration of MC. The temperature is shown on the x-axis while the endothermic heat release is shown on the y-axis. Note that the scale bar on the y-axis is not absolute and the curves have been physically separated to clarify the distinction between the various samples. Hence, no scale bar values are shown on the y-axis.

For generating a thermoresponsive hydrogel system, thermal properties of three commercially available MCs with different molecular weights (M0512, mol. wt. = 88,000; M0262, mol. wt. = 41,000; and M7140, mol. wt. = 15,000) were thoroughly investigated using a Diamond Differential Scanning Calorimeter (DSC). A typical example of calorimetric thermograms determined using a diamond DSC is shown in Figure 2 for M0512 at different concentrations of MC in iso-osmotic (0.3 M) 1× PBS solutions. In the heating process, a sharp endothermic peak was observed at around 47 °C indicating the incipient gel formation temperature. 

Thermal gelation of MC in water is caused by hydrophobic interactions between molecules containing methoxyl groups. At low temperatures, the cellulose molecules are hydrated and there is little polymer–polymer interaction apart from entanglement [34]. As the temperature is increased, molecules absorb translational energy and gradually lose their water of hydration, resulting in lowering of viscosity [35,36]. Eventually, a polymer-polymer association takes place, due to hydrophobic interactions, causing cloudiness in solution and an infinite network structure which results in a sharp rise in viscosity and turbidity as long as the concentration is relatively high [36]. Thus, the addition of MC reduces the gel formation temperature in a water-MC solution (see Table 2). However, the lower molecular weight MC (mol. wt. = 15,000) could be diluted to a maximum value of 16% (the associated gel formation temperature was 31.1 ± 1.5 °C) while the highest molecular weight MC (mol. wt. = 88,000) could only be diluted to a maximum value of 6% (the associated gel formation temperature was 48.2 ± 1.0 °C). These experiments also showed that the gel formation temperature was strongly dependent on the % of MC in solution and weakly dependent on the molecular weight of MC. Thus, further characterization experiments were performed with the low molecular weight MC, since these could be diluted the most in water.

It is interesting to note that within the molecular weight range studied there is no noticeable difference in the gelation temperature of MC solutions when compared at similar concentrations (Table 2). This is contrary to classical polymer chemistry where one correlates an increase in precipitation temperature with decrease in molecular weight [35,36]. This uncharacteristic behavior of MC solution can however be explained if one takes into account the molecular weight distribution and the state of aggregation of the polymer in solution [24,25,34,35,36]. All the methylcellulose samples have a wide molecular weight distribution where the ratio of weight to number-average molecular weight may vary from 3 to as high as 10 depending on the type of pulp used and the processing conditions [35,36]. The gelation temperatures actually reflect the influence of the high molecular weight fraction of the sample, which precipitates out first. All the samples of varying average molecular weight possibly contain similar high molecular weight fractions, although of varying amounts, thereby leading to the similar gelation temperatures between the three MC systems studied [35,36].

### 3.2. Effect of Salt Concentration and Osmolality

Our goal was to develop a MC based hydrogel that changes phase at ~32 °C and with a concentration of MC less than 14% (since, higher concentrations of MC tends to make the solution highly viscous and hard to handle). Unfortunately, as shown in Table 2, at concentrations less than 14%, the gel formation temperature of the lowest mol. wt. MC was higher than ~35 °C. Thus, we decided to investigate the effect of adding salts to the MC-water mixture. As described in prior experiments, we also found that the addition of salts lowered the gel formation temperature [24,25,26,35,37,38,39]. However, for cell culture purposes, the concentration of the salts could not (and should not) be increased above ~300 mOsm.Additionally, the osmolalities of aqueous MC solutions, used to prepare the MC hydrogels, increased nearly linearly with increasing the salt concentrations (data not shown). At higher (>1× or 300 mOsm) concentrations, the salts leached out from the hydrogel and joined the cell culture media and significantly increased its osmolality, to the detriment of the cell viability [24,40,41,42].

### 3.3. Optimal MC Concentration

**Figure 3:**
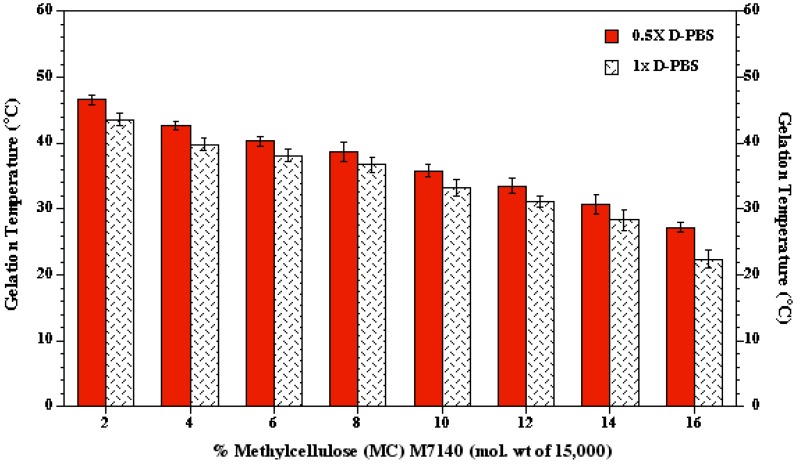
Thermal gelation temperatures of MC M7140 (Mol. wt of 15,000) in 0.5× and 1× D-PBS solutions obtained using a calorimeter. MC-PBS solutions were prepared in different concentrations by dispersing the weighed MC powders in heated D-PBS solution at 90 °C. As shown in the figure increasing the concentration of MC in solution and increasing the osmolality of the D-PBS solutions from 0.5× to 1× leads to a lower gelation temperature (n = 10). For example, a gelation temperature of ~25 °C can be achieved by using 16% MC in 1× D-PBS as opposed to a value of ~40 °C with 8% MC in 0.5× D-PBS. Since, lowering the concentration of MC leads to solutions with lower viscosity (and hence, increased ease of use), the optimal MC solution was chosen to be 14% MC with 0.5× D-PBS. This optimal solution of MC had a gelation temperature of ~32 °C. The gelation temperature is shown on the y-axis while the concentration of MC in solution (either 0.5× D-PBS or 1× D-PBS) is shown on the x-axis. The error bars represent standard deviation (n = 3).

As shown in Figure 3, the optimal combination of MC-water-salt was found to be 12% to 16% of MC (mol. wt. of 15,000) in water with 0.5× PBS (~150 mOsm). This solution exhibited a gel formation temperature of ~32 °C.

### 3.4. Effect of Culture Time—Degradation/Swelling

**Figure 4:**
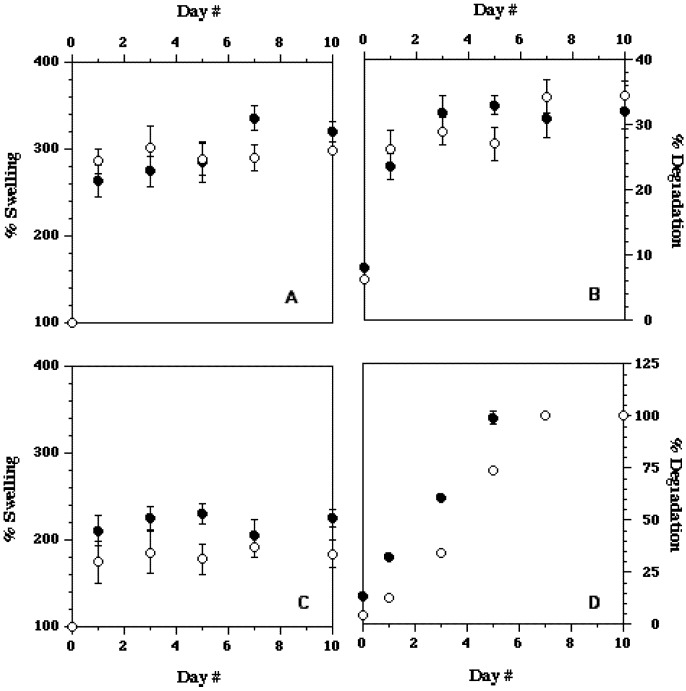
Percent swelling and percent degradation values of MC M7140 (mol. wt = 15,000) system at 14% concentration with 0.5× D-PBS as determined in our laboratory—top two figures. Percent swelling and percent degradation values of hydrogel system developed by Chen *et al.* [24]—bottom two figures. Note the different values on the y-axis between the top and bottom figures on the right. In each figure the open symbols represent data for 0.5× D-PBS while the filled symbols the corresponding data for 1× D-PBS solutions with MC. The error bars represent the standard deviations in the data (n = 3).

Further experiments to determine the stability of the MC-water-salt hydrogel with the optimal thermal/gelation parameters were also performed (Figure 4). MC hydrogels were constructed using the optimal concentration of MC (14% of M7140 blended with 0.5× and 1× PBS). The results were also compared with the hydrogel system developed by Chen *et al*. [24]. For this, 4% of M0512 was blended with 0.5× PBS and 450 µL of the solution was evenly distributed on the TCPS dish using hand. The coated TCPS dish was subsequently dried in a laminar flow hood to remove 50% of its moisture content [24]. Higher swelling rates were observed with MC hydrogel developed in our laboratory when compared to the system developed by Chen *et al*. [24] (Figure 4). Our hydrogel system also demonstrated some amount of degradation immediately after adding cell culture media (Figure 4). However, after this initial loss of polymer mass the degradation profile “leveled off” for the duration of the experiment (10 days). Conversely, the hydrogel system developed earlier [24], exhibited significant degradation for several days (Figure 4) and was also unstable; during the removal of culture media, small patches of hydrogel coating peeled off from the surface. Thus, we were reasonably confident that the hydrogel system developed in these preliminary experiments is superior to systems described in the literature [18,19,20,21,22,23,24]. 

### 3.5. Further Optimization of the Thermally Reversible Hydrogel System for ASC Culture

**Figure 5:**
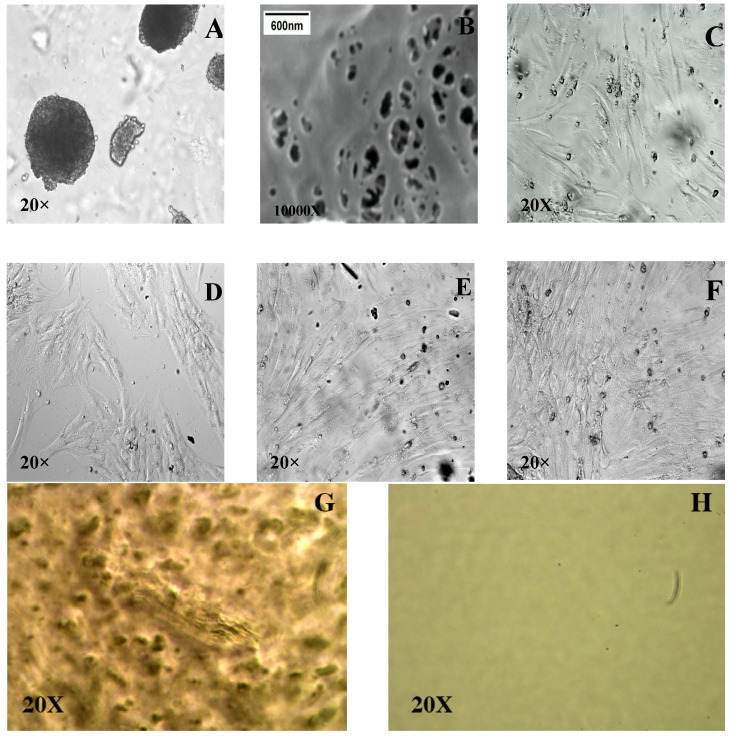
(**A**).Bright-field microscopic image ASCs grown on MC hydrogel system (with no collagen coating) for 1 day; (**B**). A Scanning Electron Microscope, SEM (JEOL 840A SEM operated at an accelerating voltage of 20 kV) image of the collagen network formed onto the MC coated TCPS dish; (**C**). Bright-field microscopic image of ASCs grown on MC hydrogel system coated with collagen for 1 day; (**D**): Bright-field microscopic image of cells attached and grown on a non-coated TCPS dish (control) for 1 day. The ASC were completely unreceptive to the hydrogel surface without collagen coating and generated embryoid-like structures (Figure 5A). The addition (evenly spread) of 200 µL of 2 mg/mL bovine collagen type -I (pH adjusted to 7.5) over the MC coated surface at 37 °C (Figure 5B), significantly improved the ASC cell adhesion on the hydrogel system (Figure 5C). The SEM image of the collagen coating (Figure 5B) confirmed the formation of an organized collagen network covering the entire surface of the MC coated TCPS dish. Bright-field microscopic image of cells grown on a control uncoated TCPS dish are also shown for reference (Figure 5D). Also shown are Bright-field microscopic images of ASCs cultured and grown for 3 days (Figure 5E) and 5 days (Figure 5F) days on the MC-hydrogel system. For reference, bright-field microscopic images of MC hydrogel (**G**) and TCPS dish (**H**) are also shown. Note that the scale bar shown in Figure 5B is relevant only to this image and the other images were obtained at a magnification of 20× (scale bar: 100 µm).

Our initial ASC culture experiments were performed by adding ~200,000 cells/mL in ASC cell culture media on the 14% MC-water-0.5× PBS hydrogel system (mol. wt. = 15,000). Unfortunately, the ASC were poorly adherent to the hydrogel surface and generated embryoid-like or spheroid-like structures, as opposed to monolayer cell sheets (Figure 5A). With the addition (evenly spread) of 200 µL of 2 mg/mL bovine collagen type -I (pH adjusted to 7.5) over the MC coated surface at 37 °C (Figure 5B), the ASC cell adhesion on the hydrogel system significantly improved (Figure 5C). Thus, we were able to grow ASC sheets on a MC-collagen hydrogel system that compares favorably with the growth response exhibited by the ASCs in an uncoated TCPS dish (Figure 5D). Figure 5 also shows the cultured ASCs at day 3 (Figure 5E) and day 5 (Figure 5F), as well; and further supports that the addition of collagen coating to MC-hydrogel system significantly improved cell adhesion and proliferation when compared to MC-hydrogel system without collagen coating (Figure 5A). 

### 3.6. Thermal Reversibility of Attachment—Effect of Temperature

**Figure 6:**
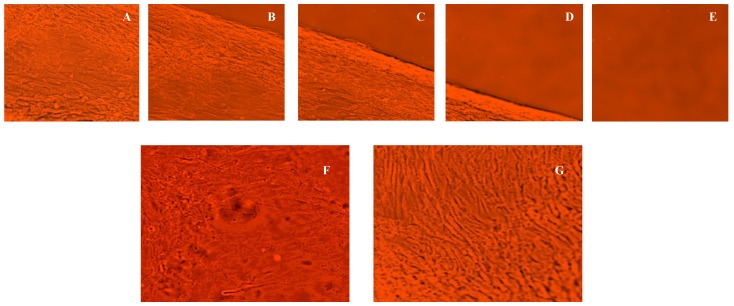
A series of bright-field microscopic images of the detaching ASC cell sheet with time (magnification 20×). The cell sheet detaches instantaneously (~1 min) after it was exposed to room temperature (~30 °C) and removed from the incubator (~37 °C) (Figure 6A–E). Figure 6F shows the monolayer of cells attached to the MC-collagen hydrogel at 37 °C while Figure 6G shows the final detached ASC sheet in suspension at ~32 °C (scale bar: 100 µm).

Upon conﬂuence, a continuous monolayer ASC sheet was formed on the surface of the MC hydrogel system (Figure 5F). When the grown cell sheet was removed from the incubator and exposed to room temperature (~30 °C), it spontaneously and gradually detached from the surface of the thermo-responsive hydrogel (without the use of detachment enzymes, like trypsin). The ASC cell sheet started detaching from its edge at ~1 min after being exposed to room temperature. Detachment of the entire cell sheet was completed within 2 to 3 min (Figure 6A–E). Additional control experiments with just a TCPS dish coated with collagen did not result in the formation and spontaneous detachment of an ASC sheet (data not shown). Figure 6F shows the image of the ASC sheet attached to the MC-collagen system at 37 °C whereas Figure 6G shows image of a detached ASC sheet obtained with the same system exhibiting the characteristics of a monolayer at ~32 °C. Since, these cells in the detached hydrogel sheet were never exposed to trypsin and other detachment enzymes, it is to be expected that the binding integrins are not compromised in the cell culture/growth/isolation process.

### 3.7. Multi-Dimensional Cell Sheets

A multilayer tissue construct was formed by using two or more MC+collagen coated TCPS dishes. P1 ASC sheets were transferred from one MC grafted culture dish as a monolayer onto another MC+collagen coated culture dish using a PVDF membrane support by lowering the temperature below 32 °C [21,43,44]. When the monolayer on MC + collagen coated dishes was incubated at a lower temperature with a donut shaped PVDF membrane over it, the polymer MC dissolved and in turn resulted in weak adhesion between the cell monolayer and the surface. The membrane together with cells was peeled off and transferred to a monolayer on another MC + collagen coated dish at 37 °C. Upon reincubation at 37 °C, cells from the membrane adhered to the monolayer as an intact cell sheet. The third cell layer was overlaid onto the double layered construct to get a three cell thick multilayered cell construct. After lowering the temperature, the multilayer cell sheet was detached generating a multidimensional cell sheet. 

The single or multilayer cell sheets developed in the present study may be used in the applications to tissue reconstructions [45,46,47,48,49,50,51,52,53,54]. Single cell sheets can be transplanted directly to host in the case of skin [18,52], corneal epithelium [47,49], and periodontal ligament [48]. By layering homotypic cell sheets in a three dimensional tissue structures and by using differentiation ability of ASCs it might be possible to create three-dimensional heterogeneous structures such as cardiac muscle [45,46]. Using heterotypic stratification, laminar structures such as kidney glomeruli, and liver lobules can also, possibly, be constructed [1].

## 4. Conclusions

A technique using a thermo-reversible MC-hydrogel coated on a TCPS dish, was developed for harvesting a living ASC sheets for tissue engineering applications. The thermal gelation properties of three commercially available MC were systematically investigated for their gel formation, swelling and degradation phenomenon at physiological temperatures. The optimal combination of MC-water-salt was found to be 12% to 16% of MC (mol. wt. of 15,000) in water with 0.5× PBS (~150 mOsm). This solution exhibited a gel formation temperature of ~32 °C. The addition (evenly spread) of 200 µL of 2 mg/mL bovine collagen type -I (pH adjusted to 7.5) over the MC coated surface at 37 °C, significantly improved the ASC cell adhesion and proliferation on the hydrogel system. Upon conﬂuence, a continuous monolayer ASC sheet was formed on the surface of the MC hydrogel system. When the grown cell sheet was removed from the incubator and exposed to room temperature (~30 °C), it spontaneously and gradually detached from the surface of the thermo-responsive hydrogel (without the use of trypsin). This non-invasive method of cell retrieval using MC coated TCPS dishes allows creation of single and multilayered cell sheet constructs preserving cell–cell and cell–extracellular matrices.
